# Urban-Rural Disparity in Cancer Incidence, Mortality, and Survivals in Shanghai, China, During 2002 and 2015

**DOI:** 10.3389/fonc.2018.00579

**Published:** 2018-12-03

**Authors:** Xiaopan Li, Yang Deng, Weina Tang, Qiao Sun, Yichen Chen, Chen Yang, Bei Yan, Yingying Wang, Jing Wang, Shuo Wang, Fan Yang, Yibo Ding, Genming Zhao, Guangwen Cao

**Affiliations:** ^1^The Key Laboratory of Public Health and Safety of Education Ministry, School of Public Health, Fudan University, Shanghai, China; ^2^Center for Disease Control and Prevention, Pudong Institute of Preventive Medicine, Fudan University, Shanghai, China; ^3^Department of Epidemiology, Second Military Medical University, Shanghai, China

**Keywords:** cancers, urban-rural disparity, incidence, mortality, survival, China

## Abstract

**Introduction:** Disparities in the incidence, mortality, and survival of cancer types between urban and rural areas in China reflect the effects of different risk factor exposure, education, and different medical availability. We aimed to characterize the disparities in the incidence, mortality, and survivals of cancer types between urban and rural areas in Shanghai, China, 2002-2015.

**Materials and Methods:** The incidence and mortality were standardized by Segi's world standard population. Trends in the incidence and mortality of cancers were compared using annual percent change. The 5-year observed and relative survivals were calculated with life table and Ederer II methods.

**Results:** Age-standardized incidences and mortalities were 212.55/10^5^ and 109.45/10^5^ in urban areas and 210.14/10^5^ and 103.99/10^5^ in rural areas, respectively. Female breast cancer and colorectal cancer occurred more frequently in urban than in rural areas, quite in contrast to liver cancer and cervical cancer. Cancers of lung and bronchus, liver, stomach, and colon and rectum were the leading causes of cancer death in both areas. Age-standardized incidence of female breast cancer and colorectal cancer in urban areas increased while gastric cancer and liver cancer decreased in both areas. Age-standardized mortalities of cancers of breast, esophagus, stomach, colon and rectum, liver, and lung and bronchus decreased in both areas. For all cancers combined, the 5-year observed and relative survivals of cancer patients were higher in urban than in rural areas. The 5-year observed and relative survivals of cancers of liver, pancreas, stomach, brain and central nervous system (CNS), and prostate were higher in urban than in rural areas. The 5-year observed and relative survivals of cervical cancer were higher in rural than in urban areas.

**Conclusions:** Factors promoting female breast cancer and colorectal cancer in urban areas and liver cancer and cervical cancer in rural areas should be specifically intervened in cancer prophylaxis. Improved medical services can greatly prolong the survival of major cancers in rural areas.

## Introduction

Cancer has been the leading cause of death in China. Data from 2013 showed that 3.7 million new diagnosed cancer cases and 2.2 million people died of cancer in mainland China (1). Approximately 22% of global new diagnosed cancer cases and close to 27% of global cancer deaths occur in China (2, 3). High levels of cancer incidence and cancer death reflect the aged society, different cancer types, increased pollution, and low level of medical services.

Since the founding of the People's Republic of China in 1949, the government has enforced a household registration system, which is different from other countries in the world. The residents are classified into two types: non-agricultural (urban) and agricultural (rural) residents. During the era of planned economy (1949-1992), urban residents mostly worked in fields of industry and commerce, purchased the necessaries of life including grain, meat, sugar, and cooking oil using their salary. Rural residents mostly lived on agricultural fields. Urban residents had the priority to enjoy some social benefits including the allotment of housing, healthcare, and education. Rural residents were usually self-sufficient and low educated (4). Urban residents experienced more industrial pollutions than rural ones while rural population had a low frequency of having refrigerators for food reservation. Since China entered the market economy era in 1993, the government has gradually eased these regulations, but rural residents still encountered barriers in obtaining basic welfares such as medical service and health education (4). Most health resources were allocated to urban residents while rural residents might not afford expensive medical expenditure, resulting in the inequity of health service utilization among urban and rural residents (5). Cancer is the second most common in rural areas and the first leading cause of death in urban areas in China (6). Data from 5 urban and 5 rural areas in the China Kadoorie Biobank cohort showed that cancer burden was different between urban and rural areas of China (7). Disparities in the incidence, mortality, and survival of cancer between the urban and rural populations may help in identifying cancer-determining socioeconomic factors that can be handled for cancer prevention and control. However, trends in the incidence and mortality of major cancer types in urban and rural areas were not fully elucidated at subnational levels in China. Moreover, there are no data interpreting the difference in cancer survival between urban and rural areas.

Shanghai is the largest metropolis in China, having 16 districts. Pudong new district has a resident population of about 5 million, accounting for 20% of the total population in Shanghai. Pudong new district was founded in 1993, and Nanhui county which represented rural areas was combined into the Pudong new district in 2009. Pudong has become the only district with urban and rural populations in Shanghai. Shanghai has established a cancer registration system since 1973 (8). Since 2002, this system has covered 100% of registered population and become one of cancer registry with the largest population worldwide. The cancer registration data are reliable and their quality has been approved by the World Health Organization. Although not exactly same, original Pudong new district and Nanhui county had equal cancer registration system. We selected Pudong new district as a suitable model to compare the disparities of cancer burden between urban and rural areas in Shanghai, which might provide evidences to optimize cancer control strategy.

## Materials and methods

### Data source

The geographic location of Pudong new district, Shanghai, China, is shown in Supplementary Figure 1. Due to time needed for data collection, quality control, and analysis, the data in this study have a 3-year time lag. The definitions of urban and rural areas in this study were based on the regulation released by the National Bureau of Statistics of China in 2006. Urban area was referred to the area with a population density of over 1,500 persons per km^2^, and rural area was the area with a population density < 1,500 persons per km^2^ (9). A total of 46 communities (32 in urban and 14 in rural) provided electronic data from 2002 to 2015 by Pudong cancer registry system and were involved in the comparison of the incidence, mortality, and survival of cancers between urban and rural areas. The detailed variables of each cancer patient including age, gender, cancer type, date of diagnosis, pathology, treatment, date and cause of death, TNM stage, and registered residence were collected. All cancers combined and 26 cancer types were identified according to the International Classification of Diseases, 10th edition (ICD-10).

Cancer cases reported to Pudong cancer registry system were followed up to check their survivals via home visits or telephone enquiries every year. The date of diagnosis of primary cancer was set as the starting point of observation, and the date of death caused by primary cancer was determined as the end-point. All information of primary cancer patients who survived from January 1, 2002 to December 31, 2015 were checked. Follow-up was finished on June 20, 2017. Population data by 5-year age group and residence were obtained from the Public Security Bureau of Pudong, Shanghai, China. The study was approved by the ethics committee of the Center for Disease Control and Prevention of the Pudong New Area, Shanghai, China.

### Quality control

The data of all cancers were checked for completeness and validity before constructing database. According to the criteria of International Agency for Research on Cancer/International Association of Cancer Registries, two authors (XL and YD) assessed the data quality independently. Three main measures including the proportion of morphological verification (MV%), percentage of cancer cases identified with death certification only (DCO%), and mortality to incidence ratio (M/I) were calculated to evaluate the data quality.

### Statistical analyses

The crude incidence and mortality rates of cancers in urban and rural areas were calculated and shown as per 100,000 (/10^5^) person-years. Age-standardized incidence rates (ASIRWs) and mortality rates (ASMRWs) by Segi's world standard population were calculated. The incidence and mortality rates between urban and rural areas were compared according to the Possion approximation method (10, 11):

(1) $$\begin{array}{r} S_{p_{1}-p_{2}}=\sqrt{\frac{X_{1}+X_{2}}{n_{1}+n_{2}}\left(1-\frac{X_{1}+X_{2}}{n_{1}+n_{2}}\right)\left(\frac{1}{n_{1}}+\frac{1}{n_{2}}\right)\;} \end{array}$$

(3) $$\begin{array}{r} \text{u}=\frac{p_{1}-p_{2}}{S_{p_{1}-p_{2}}} \end{array}$$

where the *X* in the formula A represents the number of incidence or death from a large population, and n is the sample size of this population. *p* in formula B equals to *X*/n, and it means the crude incidence or mortality rate in this population.

Cancer trends in ASIRWs and ASMRWs were calculated using Joinpoint Regression Program 4.3.1.0 (downloaded from the website of the National Cancer Institute, MD, USA) and expressed as an annual percent change (APC), and the Z test was employed to assess whether the APC was statistically different from zero. Age-specific incidence and mortality rates were calculated for each 5-year age group, from 0–4 to 85+ years. The 5-year observed survivals (OS) and relative survivals (RS) were calculated with life table and Ederer II methods (12, 13). All statistical analyses were conducted using SPSS 21.0 (SPSS, Inc., Chicago, IL) and R (version 3.4.3). *P-*value < 0.05 was considered as statistically significant.

## Results

### Incidence and mortality rates of all cancers

The average MV%, DCO%, and M/I for all cancers was 70.40%, 3.42%, and 0.56, respectively (Supplementary Table 1). These indicators suggested that overall quality of data was satisfied.

A total of 149,236 new cancer cases from 37,353,102 person-years were diagnosed during 2002-2015, with 111,139 cases from 26,870,661 person-years in urban areas and 38,097 cases from 10,482,441 person-years in rural areas. The mean ages at diagnosis were 64.73 ± 14.97 years in urban cases and 63.50 ± 15.23 years in rural cases. There were 79,223 cases from 18,667,245 person-years in men, and 70,013 cases from 18,685,857 person-years in women. The mean ages at diagnosis were 65.95 ± 13.91 years in males and 62.68 ± 16.05 years in females (Table 1). The crude incidence rates of all cancers were 413.61/10^5^ and 363.44/10^5^ in urban and rural areas, respectively. The crude incidence rate was significantly higher in urban than rural areas (*u* = 21.30, *P* < 0.01). No significant difference in ASIRW of all cancer types was found between urban (212.55/10^5^) and rural areas (210.14/10^5^) (*u* = 1.27, *P* = 0.89) (Table 2).

**Table 1:** Incidence and mortality rates of all cancers in Pudong new district, Shanghai, China, 2002-2015.

**Area**	**Gender**	**Incidence**				**Mortality**			

		***N***	**CIR**^a^	**ASIRW**^b^	**Age (years)**	***N***	**CMR**^c^	**ASMRW**^d^	**Age (years)**
All	Both	1,49,236	399.53	211.27	64.42 ± 15.04	87,668	234.70	105.44	70.84 ± 13.37
	Male	79,223	424.40	225.68	65.95 ± 13.91	53,754	287.96	138.96	70.06 ± 12.91
	Female	70,013	374.68	201.76	62.68 ± 16.05	33,914	181.50	75.35	72.07 ± 13.98
Urban	Both	1,11,139	413.61	212.55	64.73 ± 14.97	64,764	241.02	109.45	71.26 ± 13.29
	Male	58,670	435.25	223.05	66.23 ± 13.91	39,469	292.81	149.70	70.49 ± 12.91
	Female	52,469	391.82	204.93	63.05 ± 15.90	25,295	188.89	75.52	72.46 ± 13.79
Rural	Both	38,097	363.44	210.14	63.50 ± 15.23	22,904	218.50	103.99	69.65 ± 13.53
	Male	20,553	396.18	233.67	65.16 ± 13.91	14,285	275.36	135.28	68.87 ± 12.89
	Female	17,544	331.35	193.03	61.55 ± 16.44	8,619	162.79	74.79	70.94 ± 14.45

a *CIR, crude incidence rate (per 100,000)*.

b *ASIRW, age-standardized incidence rate by Segi's world standard population (per 100,000)*.

c *CMR, crude mortality rate (per 100,000)*.

d *ASMRW, age-standardized mortality rate by Segi's world standard population (per 100,000)*.

**Table 2:** Incidences of major cancer types in urban and rural areas in Pudong new district, Shanghai, China, 2002-2015.

**Rank**	**Type**	**ASIRW^a^**	**CIR^b^**	**Proportion (%)**	**APC (ASIRW) (95% CI) (%)**
**URBAN**					
1	Breast in female	39.17	69.30	8.34	2.30 (1.61, 3.12)*
2	Lung and bronchus	34.48	74.68	18.02	0.89 (−0.20, 2.05)
3	Colon and rectum	23.65	50.66	12.22	1.42 (1.21, 1.82)*
4	Thyroid	19.23	27.65	6.67	18.93 (17.10, 20.79)*
5	Stomach	19.10	40.59	9.79	−3.42 (−4.24, −2.71)*
6	Liver	13.29	26.63	6.43	−5.03 (−7.01, −3.09)*
7	Prostate	11.60	25.74	3.11	5.22 (2.92, 7.54)*
8	Cervix	10.09	14.94	1.80	9.80 (7.81, 11.92)*
9	Brain and CNS^c^	7.32	12.06	2.91	−1.84 (−3.52, −0.17)*
10	Pancreas	6.43	14.58	3.52	0.65 (−1.02, 2.20)
	All types	212.55	413.61	100.00	1.25 (0.71, 1.68)*
**RURAL**					
1	Lung and bronchus	38.46	72.49	19.74	−2.40 (−6.08, 1.47)
2	Breast in female	24.16	38.89	5.35	1.92 (−0.61, 4.42)
3	Thyroid	20.73	29.66	8.08	20.22 (12.37, 28.65)*
4	Liver	19.94	35.48	9.66	−7.02 (−11.51, −2.25)*
5	Stomach	18.74	35.08	9.55	−6.26 (−9.01, −3.27)*
6	Colon and rectum	18.64	34.46	9.38	−2.51 (−7.45, 2.62)
7	Cervix	14.77	21.16	2.91	15.50 (3.37, 29.18)*
8	Prostate	9.87	18.26	2.46	6.52 (4.73, 8.41)*
9	Pancreas	7.19	14.34	3.90	−2.30 (−4.48, −0.26)*
10	Esophagus	5.70	11.26	3.07	−6.62 (−9.78, −3.52)*
	All types	210.14	363.44	100.00	−0.92 (−2.91, 1.10)

a *ASIRW, age-standardized incidence rate by Segi's world standard population (per 100,000)*.

b *CIR, crude incidence rate (per 100,000)*.

c *CNS, central nervous system*.

* *APC value is significantly different from zero at alpha = 0.05*.

A total of 87,668 cancer deaths (53,754 men and 33,914 women) were reported from 2002 to 2015, with 64,764 in urban and 22,904 in rural areas. The mean ages at death were 71.26 ± 13.29 years in urban cases and 69.65 ± 13.53 years in rural cases. The mean age were 70.06 ± 12.91 years for men and 72.07 ± 13.98 years for women (Table 1). The crude mortality rate was higher in urban than rural areas (241.02/10^5^ vs. 218.50/10^5^, *u* = 12.58, *P* < 0.01). The ASMRW was higher in urban than in rural areas (109.45/10^5^ vs. 103.99/10^5^, *u* = −4.79, *P* < 0.01) (Table 2).

### The incidence and mortality of top 10 cancers

**Figure 1:**
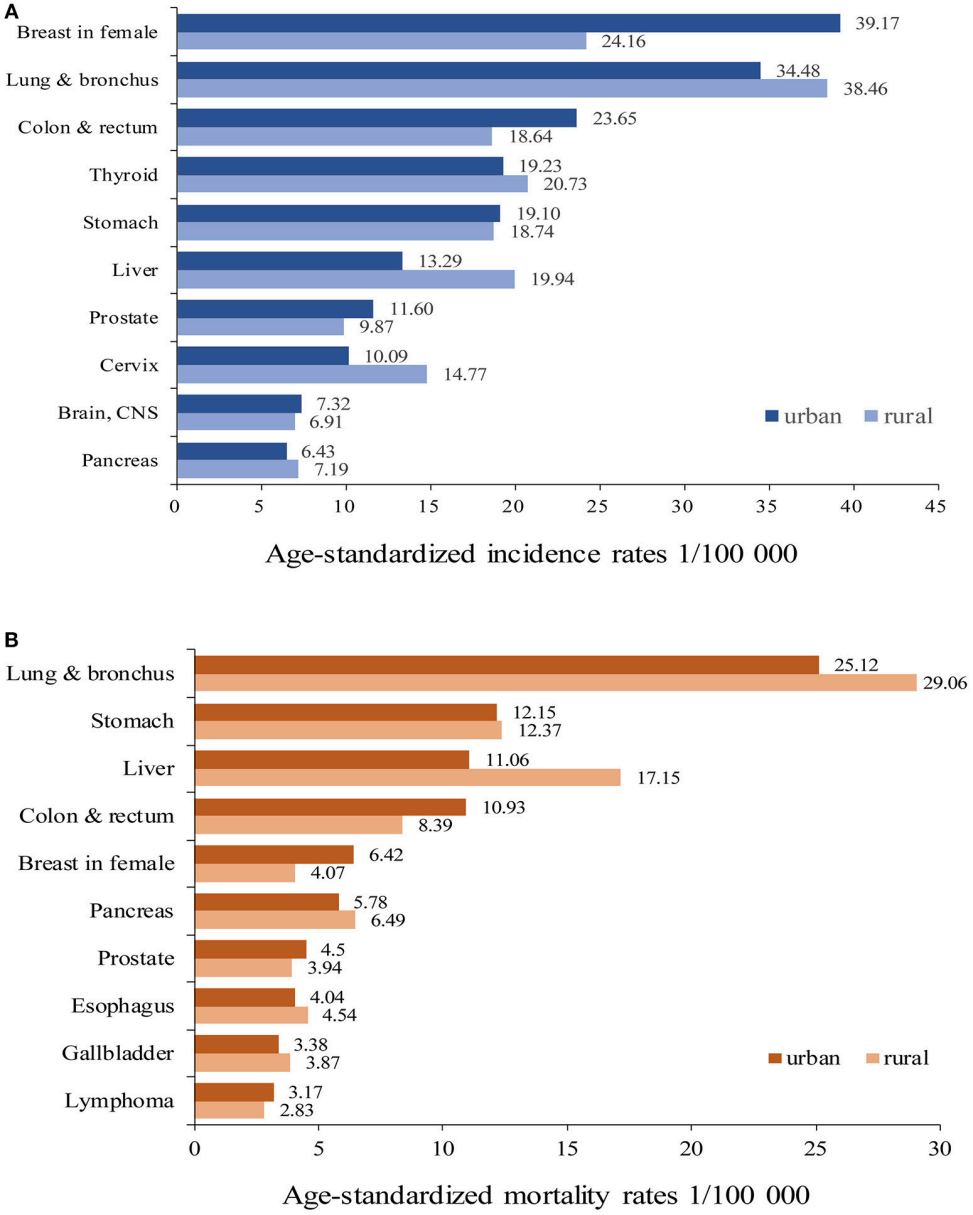
Age-standardized incidence rates **(A)** and mortality rates **(B)** of major cancer types in urban and rural areas of Pudong new district, Shanghai, China.

Cancers of breast in female, lung and bronchus, colon and rectum, thyroid, stomach, liver, prostate, cervix, brain and central nervous system (CNS), and pancreas were the top 10 cancer types in urban areas, accounting for 72.81% of all new diagnosed cancers. Cancers of lung and bronchus, breast in female, thyroid, liver, stomach, colon and rectum, cervix, prostate, pancreas, and brain and CNS were the top 10 cancers in rural areas, accounting for 74.10% of all new diagnosed cancers (Table 2). The ASIRWs of cancers of breast in female (*u* = 24.42, *P* < 0.01), colon and rectum (*u* = 20.92, *P* < 0.01), prostate (*u* = 9.57, *P* < 0.01), and brain and CNS (*u* = 3.97, *P* < 0.01) were higher in urban than in rural areas. In particular, the ASIRWs of female breast cancer and colorectal cancer (CRC) were 62 and 27% higher in urban than in rural areas, respectively. By contrast, the ASIRWs of lung and bronchus cancer (*u* = 2.55, *P* < 0.05), liver cancer (*u* = −14.61, *P* < 0.01), and cervical cancer (*u* = −8.43, *P* < 0.01) were lower in urban than in rural areas, especially with a 50% lower in liver cancer (Figure 1A).

The top 10 causes of cancer deaths were cancers of lung and bronchus, stomach, colon and rectum, liver, pancreas, breast in female, prostate, esophagus, gallbladder, and lymphoma in both areas. The proportions of top 10 cancer deaths accounted for 79.16% in urban and 81.86% in rural areas, respectively (Table 3). The ASMRWs of cancers of colon and rectum (*u* = 15.50, *P* < 0.01), breast in female (*u* = 11.45, *P* < 0.01), prostate (*u* = 6.61, *P* < 0.01) were higher in urban than in rural areas, while ASMRWs of lung and bronchus cancer (*u* = 2.08, *P* < 0.05) and liver cancer (*u* = −14.90, *P* < 0.01) were lower in urban than in rural areas (Figure 1B).

**Table 3:** Mortalities of major cancer types in urban and rural areas in Pudong new district, Shanghai, China, 2002-2015.

**Rank**	**Type**	**ASMRW^a^**	**CMR^b^**	**Proportion (%)**	**APC (ASMRW) (95% CI) (%)**
**URBAN**					
1	Lung and bronchus	25.12	57.09	24.90	−5.61 (−7.40, −3.77)*
2	Stomach	12.15	27.31	11.91	−9.12 (−12.64, −5.45)*
3	Liver	11.06	22.58	9.85	−8.54 (−10.63, −6.31)*
4	Colon and rectum	10.93	25.32	11.04	−8.44 (−13.27, −3.40)*
5	Female Breast	6.42	13.11	2.85	−15.98 (−23.43, −7.51)*
6	Pancreas	5.78	13.31	5.81	−1.90 (−4.24, 0.58)
7	Prostate	4.50	10.78	2.36	−10.12 (−16.56, −3.24)*
8	Esophagus	4.04	9.46	4.12	−9.62 (−14.27, −4.81)*
9	Gallbladder	3.38	8.06	3.51	−2.89 (−7.02, 1.65)
10	Lymphoma	3.17	6.44	2.81	−6.52 (−10.73, −2.01)*
	All types	109.45	241.02	100.00	−7.44 (−8.91, −5.95)*
**RURAL**					
1	Lung and bronchus	29.06	56.53	27.12	−7.84 (−13.25, −2.10)*
2	Liver	17.15	30.84	14.79	−10.03 (−15.34, −4.51)*
3	Stomach	12.37	24.08	11.55	−11.22 (−16.65, −5.43)*
4	Colon and rectum	8.39	16.66	7.99	−10.67 (−14.88, −6.10)*
5	Pancreas	6.49	13.19	6.33	−3.26 (−5.32, −1.15)*
6	Esophagus	4.54	9.20	4.41	−12.20 (−20.17, −3.55)*
7	Breast in female	4.07	7.09	1.72	−14.82 (−18.20, −11.34)*
8	Prostate	3.94	7.45	1.77	−7.11 (−16.27, 3.05)
9	Gallbladder	3.87	7.74	3.71	−2.48 (−5.19, 0.41)
10	Lymphoma	2.83	5.15	2.47	−7.47 (−9.72, −5.10)*
	All types	103.99	218.50	100.00	−9.45 (−11.91, −6.96)*

a *ASMRW, age-standardized mortality rate by Segi's world standard population (per 100,000)*.

b *CMR, crude mortality rate (per 100,000)*.

* *APC value is significantly different from zero at alpha = 0.05*.

### Trends in the age-standardized incidence and mortality rates

The ASIRW for all cancers combined increased by 1.25% [95% confidence interval (95% CI): 0.71–1.68%, *P* < 0.05] per year during 2002-2015 in urban areas, but it remained stable in rural areas. Among the top 10 most common cancers in urban areas, the ASIRWs increased in cancers of breast in female, colon and rectum, thyroid, prostate, and cervix and decreased in cancers of stomach, liver, and brain and CNS. In rural areas, the ASIRWs increased in thyroid cancer, cervical cancer, and prostate cancer and decreased in cancers of liver, stomach, pancreas, and esophagus (Table 2).

The ASMRW for all cancers combined decreased by 7.44% [(95% CI): −8.91 to −5.95%, *P* < 0.05] per year during 2002-2015 in urban areas and also decreased in rural areas, with an APC of −9.45% [(95% CI): −11.91% to −6.96%, *P* < 0.05] per year. Among the top 10 most common causes of cancer death, the ASMRWs for all cancers combined and cancers of breast in female, esophagus, stomach, colon and rectum, liver, lung and bronchus, and lymphoma decreased in both areas. Decreases in ASMRWs of prostate cancer in urban and pancreatic cancer in rural areas were also evident (Table 3).

### Age-specific incidence and mortality

**Figure 2:**
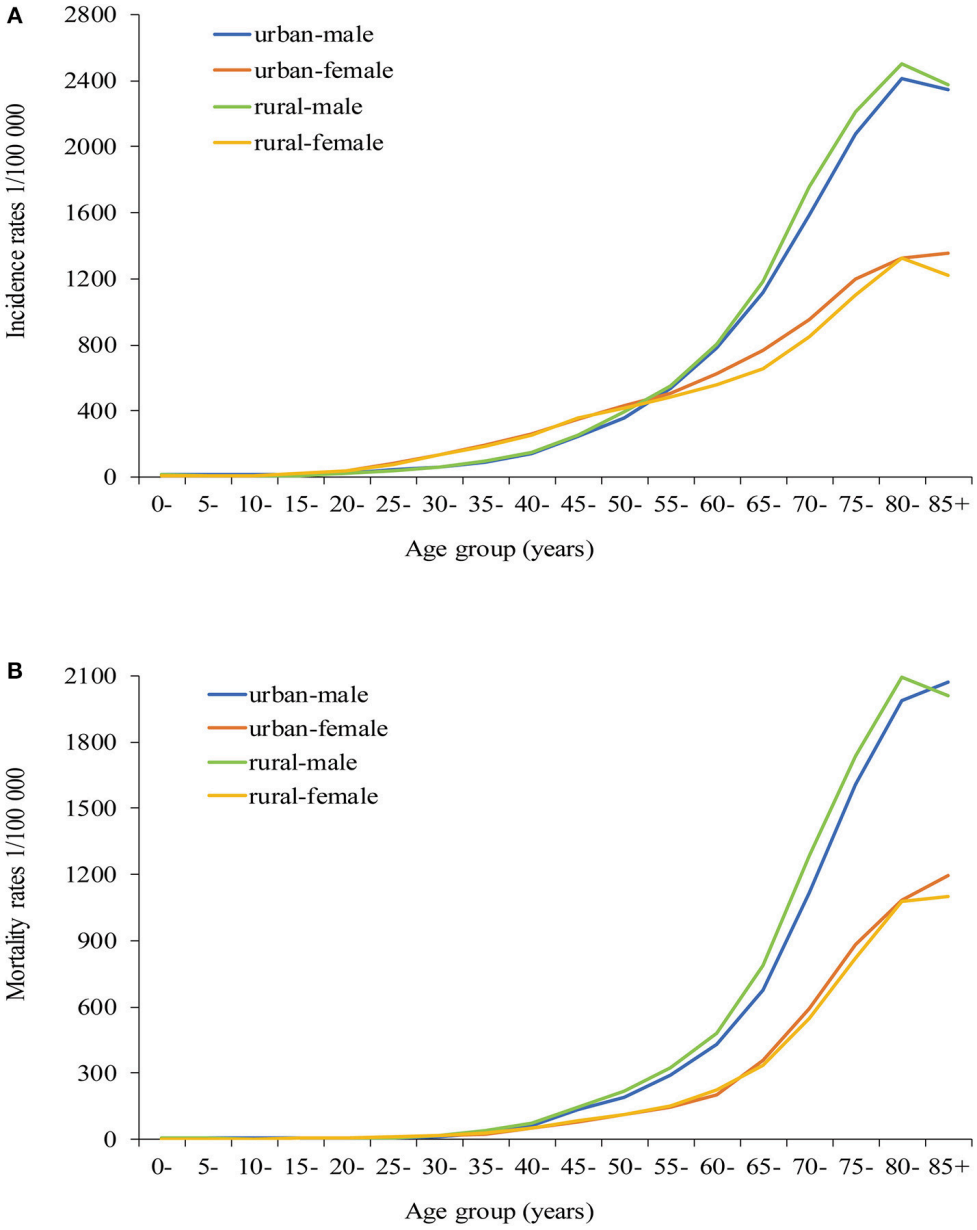
Age-specific incidence rates **(A)** and mortality rates **(B)** of all cancer types in urban and rural areas of Pudong new district, Shanghai, China.

The age-specific incidence rates were lower in men than in women aged 30–54 years, while a rapid increase was observed in men. Among those older than 55 years, the incidence rates were consistently higher in men than in women. In rural areas, the incidence rate increased slowly from 30 to 59 years and increased rapidly after 60 years, reaching the peak after 80 years in men, while the incidence increased continuously from 30 years and peaked after 80 years in women. The pattern was similar in rural areas, and a more significant increase was observed in men older than 60 years (Figure 2A).

The age-specific mortality rates increased slowly from 30 to 59 years, after which the rates increased rapidly in men. However, the age of switch occurred between 65 and 70 years in women. The mortality rates of all age groups were always higher in rural than in urban areas in men. Compared with urban areas, no significant difference in mortality rates was observed in rural areas in women (Figure 2B).

### Five-year OS and RS of major cancers in urban and rural areas

For all cancers combined, the 5-year OS of cancer patients was higher in urban than in rural areas (44.05 vs. 41.47%, *P* < 0.001), and the 5-year RS was 4.76% higher in urban areas than in rural areas. Cancers of pancreas, gallbladder, liver, esophagus, and lung and bronchus had the poorest 5-year OS and RS in both areas (Table 4).

**Table 4:** Five-year observed and relative survivals for cancer patients diagnosed in urban and rural areas in Pudong new district, Shanghai, China, 2002-2015.

**Type**	**Cases**		**5-year OS**^a^			**5-year RS**^b^		

	**Urban**	**Rural**	**Urban**	**Rural**	***P***	**Urban**	**Rural**	**Difference in RSR (Urban-Rural)**^c^
Lung and bronchus	19,909	7,503	18.07	16.53	0.613	25.47	22.67	2.80
Liver	7,354	3,834	14.37	11.81	< 0.001	18.83	14.98	3.85
Pancreas	3,954	1,498	5.91	5.03	0.004	8.98	7.63	1.35
Gallbladder	2,531	955	11.81	12.86	0.109	18.78	19.64	−0.86
Esophagus	3,168	1,198	17.17	16.08	0.149	25.53	23.52	2.01
Stomach	11,076	3,741	32.65	30.52	0.037	45.62	41.46	4.16
Colon and rectum	13,579	3,586	50.16	52.16	0.284	70.00	70.29	−0.29
Lymphoma	2,831	890	38.77	40.24	0.253	49.95	51.52	−1.57
Brain and CNS^d^	3,318	1,129	56.10	54.51	0.045	69.69	65.82	3.87
Breast in female	9,320	2,048	83.74	83.48	0.534	95.52	92.87	2.65
Cervix	1,965	1,069	83.74	87.22	0.002	90.58	91.84	−1.26
Prostate	3,350	902	59.20	53.93	0.022	96.58	90.02	6.56
Thyroid	6,793	2,768	95.88	96.88	0.209	101.63	101.81	−0.18
All types	1,11,139	38,097	44.05	41.47	< 0.001	58.44	53.68	4.76

a *OS, observed survival*.

b *RS, relative survival*.

c *Positive number indicates a higher survival in urban areas*.

d *CNS, central nervous system*.

Cancer types whose 5-year OS were higher in urban than in rural areas were liver cancer (14.37 vs. 11.81%, *P* < 0.001), pancreatic cancer (5.91 vs. 5.03%, *P* = 0.004), gastric cancer (32.65 vs. 30.52%, *P* = 0.037), brain and CNS cancer (56.10 vs. 54.51%, *P* = 0.045), and prostate cancer (59.20 vs. 53.93%, *P* = 0.022). The 5-year OS of cervical cancer was higher in rural than in urban areas (83.74 vs. 87.22%, *P* = 0.002). Cancers of liver, pancreas, stomach, brain and CNS, and prostate had higher 5-year RS in urban than in rural areas, while the cervical cancer had lower 5-year RS in urban than in rural areas (Table 4).

## Discussion

In this study, we analyzed urban-rural disparity in cancer burden in Shanghai, China in the past 14 years and confirmed that female breast cancer and CRC occurred more frequently in urban than in rural populations, in contrast to liver and cervical cancers. In 1993, Pudong was set as an Open Economic Zone. Economy has been growing fast. Lifestyle of urban residents has turned to be more westernized, with unhealthy diets and insufficient physical activity. Overweight and obesity, the risk factors for female breast cancer (14), became an increasing public health concern (15, 16). Increasing incidence of female breast cancer is partially attributed to menstrual and reproductive factors such as earlier age at menarche, later ages at menopause and first birth, fewer number of live births, and less duration of breastfeeding (17). The Chinese birth control policy enforced since early 1970s not only limited urban couples to one child and rural couples to two children but also encouraged late marriage and childbearing (18). Compared with rural women, urban women had few number of live births, later ages at birth, earlier ages at menarche, less average duration of breastfeeding, later ages at menopause (19). We showed here that the incidence of female breast cancer increased by 2.3% annually in urban areas during study period. However, some urban women who were affected by this policy have not reached the peak age of disease occurrence (45–55 years) until the end of this study. We speculate that the incidence of female breast cancer will keep increasing. The government has provided free breast cancer screening to rural residents and low-income women since late 2000s, but the coverage rate was only 27.4% in 2010 (20). Our results indicated that the mortality of female breast cancer significantly declined in urban and rural areas, possibly because of the improved diagnosis and treatment, rather than screening (21). Thus, effective and affordable breast cancer prevention and control strategies are urgently needed.

We found that the incidence and mortality of CRC were higher in urban than rural areas. The incidence of CRC increased by 4.2% annually in Shanghai in the past 30 years, which almost reached the incidence level in developed countries (2, 22). The risk factors of CRC are also related to western lifestyles. High red and processed meat intake, overweight and obesity, low vegetable and fruit intake, tobacco smoking, alcohol drinking, and physical inactivity are major risk factors for CRC (14, 23). Compared with rural residents, urban residents are more exposed to these risk factors (24). Moreover, urban residents are more likely to participate screening of CRC, and improved diagnosis and screening are partially responsible for the higher rates in urban areas. The incidence of CRC increased in urban areas and kept steady in rural areas, but the mortality decreased in urban and rural areas. Shanghai government launched a community-based CRC screening program including 542,430 urban and 267,098 rural residents. A total of 1,630 CRC patients were newly diagnosed, and 51.6% of CRC cases were diagnosed in early stage, which is equal to five times the rate of cancer registry data (22). CRC screening is effective for early diagnosis and treatment, and ultimately reduce the mortality of CRC (25). Thus, the improvement in lifestyle including increased physical activity, health food consumption, and screening are effective in reducing CRC-related death.

The incidence and mortality of liver cancer declined significantly in urban and rural areas. Chronic infection with hepatitis B virus (HBV) and/or hepatitis C virus (HCV), heavy alcohol use, and excessive aflatoxin exposure increase the risk of liver cancer (26–28). After national HBV vaccination in newborns in China from 1992, the prevalence rate of hepatitis B surface antigen (HBsAg) among nationwide population decreased from 9.8% in 1992 to 7.18% in 2006 (29–31). The vaccination rate of HBV in newborn reached over 95% in urban areas and 80% in most rural areas in 2006, thus contributing to the decreased rate of liver cancer (32, 33). The incidence and mortality of liver cancer were significantly higher in rural residents than in urban residents in this study, possibly because HBsAg prevalence was higher in rural than in urban areas (29). Currently, there are 94 million chronic HBV carriers in China. How to provide effective and affordable prophylactic options to prevent the occurrence of liver cancer in rural HBV-infected population is a great challenge.

Interestingly, the incidence of thyroid cancer and cervical cancer increased dramatically in both areas. For thyroid cancer, over-diagnosis due to widespread use of screening technologies including ultrasound can explain the dramatic increase (34, 35). The incidence of cervical cancer increased by 9.8 and 15.5% annually in urban and rural areas, respectively. High-risk human papilloma virus (HR-HPV) infection due to multiple sexual partners has been positively associated with the occurrence of cervical cancer (36–38). China has experienced an epidemic of sexual transmitted diseases since reform and opening up in the late 1970s, resulting in an increase in HR-HPV infection. The prevalence of HR-HPV was higher in rural (18.0%) than in urban areas (15.2%), and more rural women had multiple sexual partners (22%) than urban women (16%) (37). Furthermore, ages at the first intercourse and first birth of rural women were 3–4 years earlier than did urban women (36). The Chinese government has provided free cervical cancer screening for 10 million rural women per year since 2009, and HPV vaccine that can reduce the risk of cervical cancer has been available in some big cities in China (37). The increased occurrence of thyroid cancer reflect the profit' seeking medical behaviors-caused over-diagnosis. The increased occurrence of cervical cancer reflect the need of public health education in rural populations.

The socioeconomic factors have important impacts on the urban-rural disparities in cancer survival. Tertiary hospitals, which have ability to provide early detection and effective treatment for cancer, are mostly located in urban areas. In rural areas, poor cancer care and limited access to tertiary hospitals reduces the opportunity, hence the patients are often diagnosed at late stages and receive ineffective treatments in township health centers (4). The urban-rural economic disparity is obvious in China. For example, the annual per capita disposable income of residents was $4,739 and $1,600 in urban and rural households in 2014. The high out-of-pocket expenditure of anticancer drugs may discourage treatments for cancer patients in rural areas. For instance, the average annual medical cost for patients with lung cancer was $11,566 during 2002-2006, which exceeded the financial ability to pay in rural households (39). In order to ensure equal access to the high-quality health care system for all residents, the Chinese government implemented the Urban Employee Basic Medical Insurance (UEBMI) in 1998 and the Urban Resident Basic Medical Insurance (URBMI) in 2007 among urban residents, and the New Rural Cooperative Medical Scheme (NRCMS) in 2003 among rural residents (40). The coverage rates of three main health care systems (UEBMI, URBMI, and NRCMS) have increased to 89% among urban residents and 97% among rural residents by 2012. However, the reimbursement ratios of hospitalized patients covered by the UEBMI (74.64%) and URBMI (59.23%) were significantly higher than patients under the NRCMS (48.04%) (41). More investments are needed to reduce the disparity in health care and develop an equal medical insurance system for urban and rural residents.

Our study have several limitations. First, difference in exposure to risk factors including socioeconomic state, smoking, alcohol use, dieting habit, and chronic infection with HBV, HCV, and HPV between the urban and rural populations are important to characterize the controllable causes of cancer types. However, these data were unavailable. Second, our study spanned a relative short time period of 14 years (2002-2015), and further researches are needed to assess the long-term disparities in cancer burden between urban and rural areas.

In conclusion, female breast cancer and CRC occurred more frequently in urban than in rural areas, quite in contrast to liver cancer and cervical cancer. Cancers of lung and bronchus, liver, stomach, and colon and rectum were the leading causes of cancer death in both areas. Age-standardized incidence of female breast cancer and CRC in urban areas increased while gastric cancer and liver cancer in both areas decreased. Age-standardized mortalities of cancers of the breast, esophageal, gastric, colon and rectum, liver, and lung and bronchus decreased in both areas. The 5-year survivals of patients with major cancers were higher in urban than in rural areas. Thus, factors promoting female breast cancer and CRC in urban areas and liver cancer and cervical cancer in rural areas should be specifically intervened in cancer prophylaxis. Our results may provide evidence to optimize cancer control strategy.

## Author contributions

XL and YD drafted the manuscript, and participated in the collection, analysis and interpretation of data. WT, QS, YC, CY, BY, YW, JW, SW, FY, YD, and GZ contributed to data collection, suggestion for analysis. GC conceived of the study, and participated in its design and coordination and helped to draft the manuscript. All authors read and approved the final manuscript.

### Conflict of interest statement

The authors declare that the research was conducted in the absence of any commercial or financial relationships that could be construed as a potential conflict of interest.
