# Supplementary material for: Urban-Rural Disparity in Cancer Incidence, Mortality, and Survivals in Shanghai, China, During 2002 and 2015
Source: Front Oncol. 2018 Dec 3;8:579. doi: 10.3389/fonc.2018.00579 (PMC6287035; doi:10.3389/fonc.2018.00579)
Supplement: Supplementary file 1 [file Table_1.docx]

***Supplementary Materials***

**Urban-Rural** **Disparity in Cancer Incidence, Mortality, and Survivals in Shanghai, China, During 2002 and 2015**

**Xiaopan Li ^†^, Yang Deng ^†^, Weina Tang ^†^, Qiao Sun, Yichen Chen, Chen Yang****, Bei Yan, Yingying Wang, Jing Wang, Shuo Wang, Fan Yang, Yibo Ding, Genming Zhao^*^, Guangwen Cao^*^**

**^*^ *Correspondence:*** *Guangwen Cao: gcao@smmu.edu.cn.*

*Genming Zhao: gmzhao@shmu.edu.cn.*

**
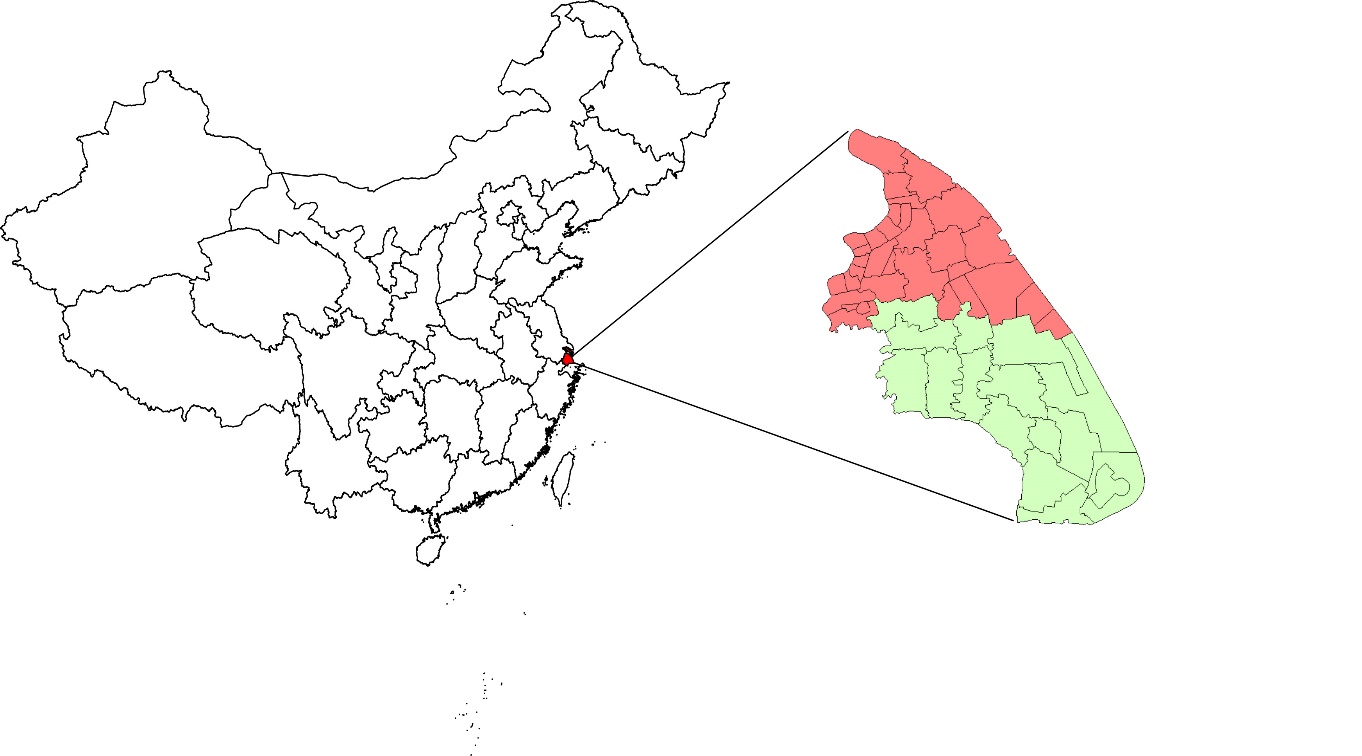
**

**Supplementary Figure 1** | The geographic location of Pudong New Area in China.

Urban areas are presented in red color; rural areas are presented in green color.

**Supplementary Table 1** | The three main measures of data quality for cancer registry in Pudong new district, Shanghai, China, 2002-2015

| **Type** | **ICD-10** | **MV%^a^** | **DCO%^b^** | **M/I^c^** |
| --- | --- | --- | --- | --- |
| Lip, oral cavity, & pharynx (except nasopharynx) | C00-C10, C12-C14 | 88.47 | 1.86 | 0.44 |
| Nasopharynx | C11 | 82.38 | 1.99 | 0.47 |
| Esophagus | C15 | 67.16 | 6.46 | 0.82 |
| Stomach | C16 | 75.59 | 3.38 | 0.68 |
| Colon & rectum | C18-C20 | 81.73 | 1.75 | 0.50 |
| Liver | C22 | 55.38 | 6.86 | 0.86 |
| Gallbladder | C23-C24 | 42.37 | 4.76 | 0.87 |
| Pancreas | C25 | 56.62 | 6.08 | 0.92 |
| Larynx | C32 | 89.50 | 2.33 | 0.43 |
| Lung & bronchus | C33-C34 | 51.41 | 4.80 | 0.77 |
| Other thoracic organs | C37-C38 | 53.08 | 3.58 | 0.58 |
| Bone | C40-C41 | 49.28 | 12.05 | 0.68 |
| Melanoma of the skin | C43 | 84.36 | 2.47 | 0.60 |
| Breast in female | C50 | 92.36 | 0.71 | 0.19 |
| Cervix | C53 | 94.25 | 0.45 | 0.14 |
| Uterus | C54-C55 | 90.68 | 1.53 | 0.22 |
| Ovary | C56 | 80.89 | 1.62 | 0.47 |
| Prostate | C61 | 77.79 | 1.31 | 0.42 |
| Testis | C62 | 85.16 | 1.10 | 0.20 |
| Kidney | C64-C66, C68 | 77.19 | 1.66 | 0.34 |
| Bladder | C67 | 82.78 | 1.98 | 0.41 |
| Brain & CNS^d^ | C70-C72 | 57.88 | 4.04 | 0.47 |
| Thyroid | C73 | 95.44 | 0.24 | 0.04 |
| Lymphoma | C81-C85, C88, C90, C96 | 71.11 | 3.31 | 0.61 |
| Leukemia | C91-C95 | 39.94 | 4.98 | 0.71 |
| All other sites and unspecified | A_O | 60.91 | 4.31 | 0.52 |
| All types | ALL | 70.40 | 3.42 | 0.56 |

*^a^MV, morphological verification.*

*^b^DCO, Death certification only**.*

*^c^M/I, mortality to incidence ratio.*

*^d^CNS, central nervous system.*
